# The wing pattern of *Moerarchis* Durrant, 1914 (Lepidoptera: Tineidae) clarifies transitions between predictive models

**DOI:** 10.1098/rsos.161002

**Published:** 2017-03-01

**Authors:** Sandra R. Schachat

**Affiliations:** 1 Mississippi Entomological Museum, Mississippi State University, Mississippi State, MS 39762, USA; 2 Department of Paleobiology, Smithsonian Institution, MRC 121, Washington, DC 20013, USA

**Keywords:** colour pattern, development, homology, morphology, scales

## Abstract

The evolution of wing pattern in Lepidoptera is a popular area of inquiry but few studies have examined microlepidoptera, with fewer still focusing on intraspecific variation. The tineid genus *Moerarchis* Durrant, 1914 includes two species with high intraspecific variation of wing pattern. A subset of the specimens examined here provide, to my knowledge, the first examples of wing patterns that follow both the ‘alternating wing-margin’ and ‘uniform wing-margin’ models in different regions along the costa. These models can also be evaluated along the dorsum of *Moerarchis*, where a similar transition between the two models can be seen. Fusion of veins is shown not to effect wing pattern, in agreement with previous inferences that the plesiomorphic location of wing veins constrains the development of colour pattern. The significant correlation between wing length and number of wing pattern elements in *Moerarchis australasiella* shows that wing size can act as a major determinant of wing pattern complexity. Lastly, some *M. australasiella* specimens have wing patterns that conform entirely to the ‘uniform wing-margin’ model and contain more than six bands, providing new empirical insight into the century-old question of how wing venation constrains wing patterns with seven or more bands.

## 1. Introduction

During recent decades, the longstanding scientific interest in the evolution of lepidopteran wing pattern has experienced a revival [1]. Though the vast majority of studies have focused on butterflies, various contributions have recognized the importance of broader evolutionary context, including the wing patterns of microlepidoptera [2,3]—a paraphyletic grade of early-diverging Lepidoptera often known as ‘tiny brown moths.’ Attempts to designate homologous wing pattern elements between butterflies and microlepidoptera began over a hundred years ago [4], but this issue remains unresolved—largely because homologies within microlepidoptera are still not fully understood.

**Figure 1.**
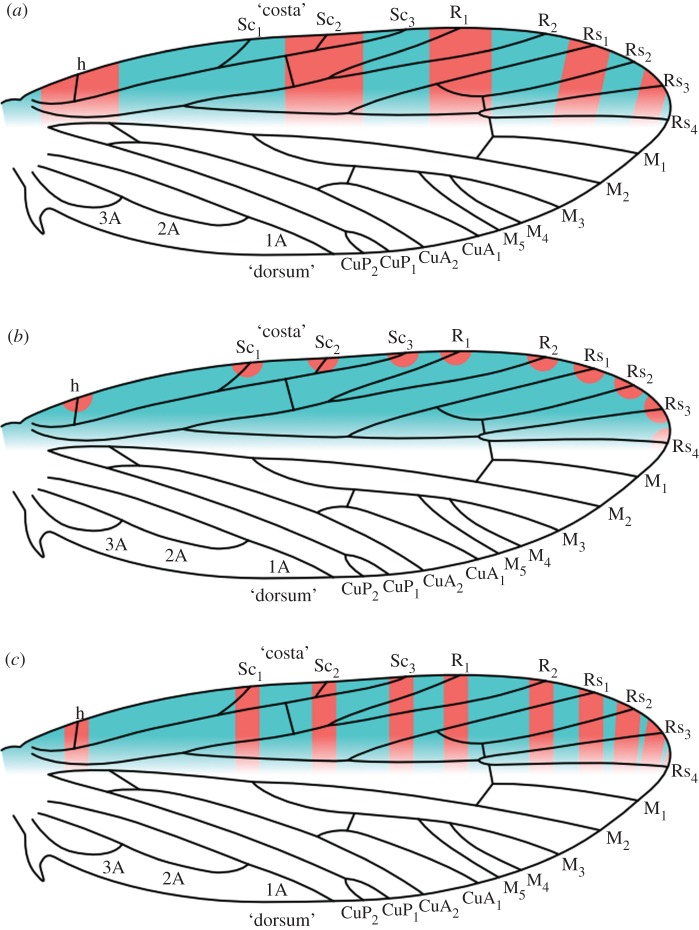
The two versions of the ‘wing-margin’ model, plotted onto the most recent reconstruction of ancestral wing venation for Lepidoptera [14]. The boundary between the costa and dorsum is ambiguous here; the Rs_4_ vein is treated as belonging to the costa because of the developmental constraints that it exerts in the Micropterigidae [10,11]. Either series of pattern elements—those illustrated in blue, or those illustrated in red—could develop a dark colour. The bands are not shown to reach the dorsum here, despite the fact that wing pattern does extend to the dorsum, because the ancestral relationship between the costa and dorsum is not yet known for banded wing patterns. (*a*) The original version of the model, called the ‘alternating wing-margin’ model here. (*b*) A hypothesized intermediate stage, based on observations of *Sabatinca demissa* [11]. (*c*) The ‘uniform wing-margin’ model.

One century ago, two studies examined the relationship between wing pattern and wing venation in individual genera and families of microlepidoptera, but did not propose any predictive models that could be tested explicitly in the context of ordinal-level homologies [5,6]. The nymphalid ground plan, a model for wing pattern in butterflies, was proposed soon thereafter, in the 1920s [7,8]. A decade later, in 1935, the first predictive model for wing pattern in microlepidoptera was proposed [9]. This model, now called the ‘vein-fork’ model, predicts that the basal edge of each dark band lies along the points where veins bifurcate; recent studies have found no support for this model [10,11]. A second predictive model for microlepidopteran wing patterns—previously known simply as ‘wing-margin’ model, and called the ‘alternating wing-margin’ model here—was proposed much more recently [12,13]. According to this model, dark and light bands straddle/abut alternating veins along the costal margin of the forewing (figure 1*a*); two recent studies strongly support this model [10,11].

The ‘vein-fork’ model predicts a primitive wing pattern ground plan with seven dark bands, whereas the ‘alternating wing-margin’ model predicts either five or six dark bands, depending on whether Rs_4_ terminates along the costa or the termen. Though Lemche’s ‘vein-fork’ model appears to have little predictive power, he was correct in recognizing the need to understand wing patterns with more than six dark bands, as such wing patterns do occur in various taxa [5,15]. A recent examination of the micropterigid genus *Sabatinca* suggested a mechanism through which patterns with more than six bands could arise from ancestral patterns that follow the ‘wing-margin’ model: dark spots accumulate around all veins at the costa—rather than alternating veins—as seen in derived *Sabatinca* species such as *Sabatinca demissa* (figure 1*b*), and these spots can eventually extend down towards the dorsum, forming bands (figure 1*c*). This newest model is now called the ‘uniform wing-margin’ model, and the wing patterns of various Psychidae conform to its predictions [16].

Because so few lineages of microlepidoptera have been examined in this context, many questions remain. The relative prevalence of the ‘alternating wing-margin,’ ‘uniform wing-margin,’ and other potential models are unknown; the transition between the two variants of the ‘wing-margin’ model is still not understood; and the relationship between wing pattern and wing venation along the dorsal margin of the wing has not yet been explored.

Psychidae and Tineidae are the two most early-diverging moth families that contain over 1000 described species [17], and these two families occupy an intermediate phylogenetic position between the Micropterigidae that have been examined very recently [10,11] and the apoditrysian moths in the family Tortricidae that originally inspired the ‘alternating wing-margin’ model [12,13]. The wing patterns of various Psychidae were described in a recent publication [16].

The tineid genus *Moerarchis* contains seven species [18]. Of these, the two Australian species *Moerarchis australasiella* (Donovan, 1805) and *Moerarchis clathrata* (Felder and Rogenhofer, 1875) have highly variable wing patterns comprising many spots and bands. Each wing pattern always consists of two colours—very light brown markings against a very dark brown background—but the number, shape and size of the individual pattern elements varies greatly between individuals. The species-level taxonomy of *Moerarchis* requires extensive further study [19], but recent systematic work has confirmed that *Moerarchis* occurs within the ‘tineine lineage’ [20].

**Figure 2.**
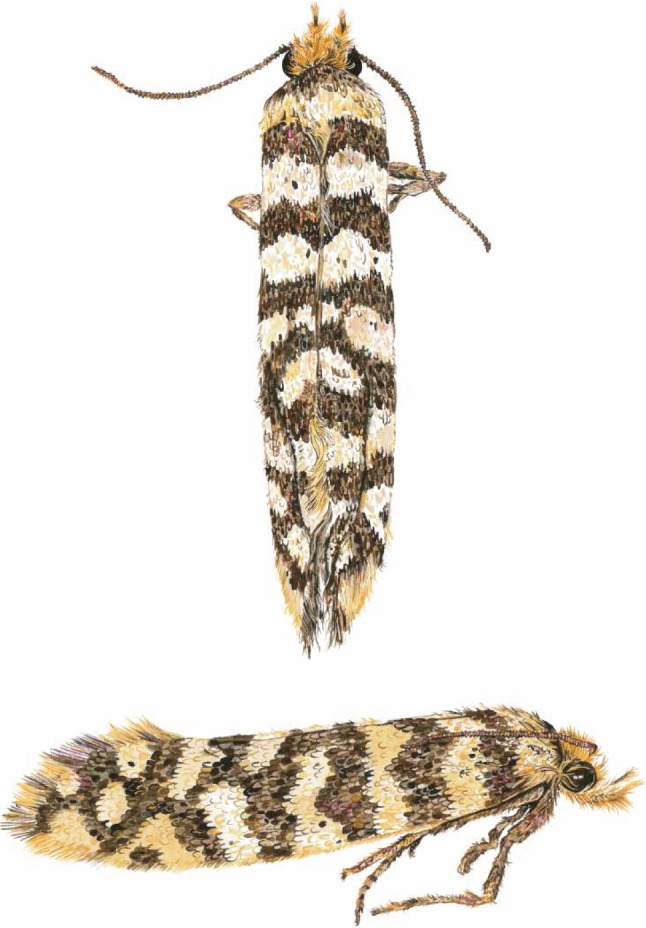
Watercolour illustrations showing two views of the same specimen, belonging to the species *Moerarchis australasiella*. Courtesy of Celia L. Curtis.

Tineidae are not generally considered to be colourful, but *Moerarchis* is very charismatic (figure 2) and has been noted for its ‘striking’ wing patterns [21]. *Moerarchis australasiella* was even featured as adornment on the cover of *Tineid Genera of Australia (Lepidoptera)* [19] and is one of three insect species included in the logo of the Australian National Insect Collection. *Moerarchis australasiella* was also the first tineid species described from Australia [18]. The high intraspecific variation of wing pattern in *Moerarchis* provides an opportunity to explore the evolution of this character in microlepidoptera.

## 2. Material and methods

All specimens examined here belong to the species *M. clathrata* and *M. australasiella* and are held in the Australian National Insect Collection in Canberra, Australia. All ‘pattern elements’ discussed here are the very light brown markings that occur on the wing. Only forewings were examined because hindwings do not have any colour pattern. All pinned specimens with spread wings were initially examined. *Moerarchis clathrata* and *M. australasiella*, and the two sexes within each species, are represented in different proportions in the Australian National Insect Collection, and so different numbers of specimens were examined for each of these groups.

The only specimens that were not examined were those whose right and left forewings are both badly damaged, owing to either wear or breakage. For each specimen that was suitable for examination, one forewing was photographed. If both wings were in equally suitable condition, the right forewing was photographed. Only unique wing patterns were illustrated; if a specimen was found to have the same wing pattern as another specimen that had already been figured, then its wing pattern was not illustrated. One hundred and eighty two specimens were examined and 159 unique wing patterns were illustrated.

To observe the relationship between wing pattern and wing venation, the clearing agent Histolene was applied to individual forewings. Because Histolene leaves a residue that can only be removed by very careful application of acetone, this method may be destructive in some cases and, therefore, could only be carried out on a subset of the specimens examined. Twenty individuals, five representatives of each sex and species, were chosen with the goal of examining the widest possible variety of wing patterns. When Histolene was applied, both the right and left forewings were examined, in order to illustrate variation within individuals.

To produce all illustrations, of wing pattern alone and of pattern and venation together, initial photographs were taken with a Leica DSC 500 microscope/camera and the Leica Application Suite software. Illustrations were created by tracing the light and dark pattern elements in each photo using Affinity Designer graphics software. Each specimen was assigned a unique number that begins with the letter ‘S.’

To explore the relationship of wing pattern with wing length, the wing patterns of each sex and species were divided into two categories (‘joined’ and ‘spotted’ for *M. clathrata*, and wing patterns with ‘few’ or ‘many’ discrete pattern elements for *M. australasiella*). Ten specimens representing each category were randomly selected for both sexes of *M. clathrata*, and the 10 specimens with the most and fewest discrete pattern elements were selected for both sexes of *M. australasiella* (electronic supplementary material, table S1). One wing of each specimen was measured in its longest dimension. Potential differences in wing length for each pair of wing pattern categories were tested with a Mann–Whitney–Wilcoxon test in R [22] using the base function wilcox.test.

## 3. Results

The wing patterns of *M. australasiella* consist of common pattern elements that can usually be recognized on wings belonging to different individuals. Twenty-three specimens belonging to *M. australasiella* have wing patterns that are not unique, and had already been illustrated from other specimens. The wing patterns of *M. clathrata* were more variable and were assigned to one of two categories: ‘joined’ wing patterns consist of many light spots and bands that are individuated at the costa and/or dorsum, but often become confluent in the interior of the wing, and ‘spotted’ wing patterns primarily contain spots. ‘Joined’ and ‘spotted’ wing patterns often appear very similar, causing the distinction between the two to be unclear in many cases; these categories can therefore be considered end points of a single continuum. All wing patterns of *M. clathrata* were found to be unique.

The 159 unique wing patterns are as follows: 40 wing patterns from female *M. australasiella* (electronic supplementary material, figures S1–S4), 61 from male *M. australasiella* (electronic supplementary material, figures S5–S9), 27 from female *M. clathrata* (electronic supplementary material, figures S10–S12) and 31 from male *M. clathrata* (electronic supplementary material, figures S13–S15).

Wing pattern elements are as finely differentiated along the dorsum as they are along the costa. All wings of both species have a large spot of light scales at the base of the wing that nears or reaches the costa and reaches the dorsum. Immediately distal to this large spot is an unbroken light transverse band that reaches the dorsum and either approaches or reaches the costa, with very few exceptions: this band is broken in one specimen (electronic supplementary material, figure S9*l*) and does not reach the dorsum in two specimens (electronic supplementary material, figures S10*a* and S15*j*). In *M. clathrata*, this band can become partially confluent with other pattern elements. The wings of *M. australasiella* have between four and eight differentiated patches of light colour (separated by patches of dark colour) along the costa, and between six and 10 along the dorsum, including the spot at the base of the wing. Many pattern elements are transverse bands that extend from the costa to the and dorsum, and many pattern elements that reach the same wing margin become confluent in the interior of the wing.

**Figure 3.**
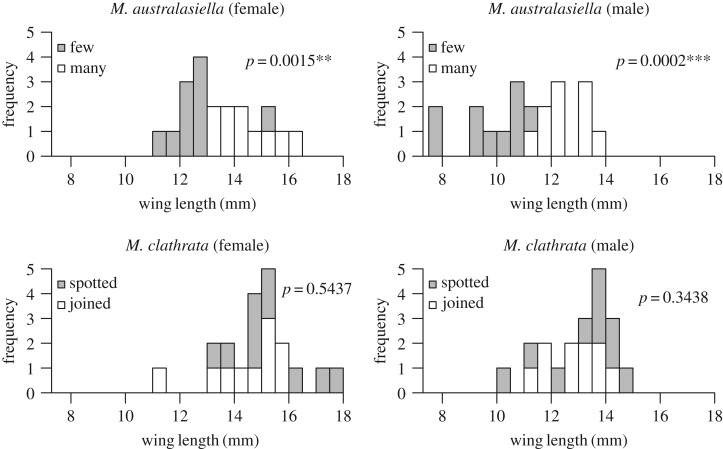
Comparison of wing length among specimens with different wing patterns.

The differences in wing length between *M. clathrata* specimens with ‘spotted’ and ‘joined’ colour patterns are not significant; however, *M. australasiella* specimens with longer wings tend to have significantly more pattern elements on the wing in both sexes (electronic supplementary material, table S2; figure 3). The wings of males tend to be smaller than those of females (figure 3).

### 3.1. Wing pattern and wing venation

In the species examined here, six veins reach the costa: Sc, R, Rs_1_, Rs_2_, Rs_3_ and Rs_4_. Rs_4_ often terminates near the apex of the wing.

**Figure 4.**
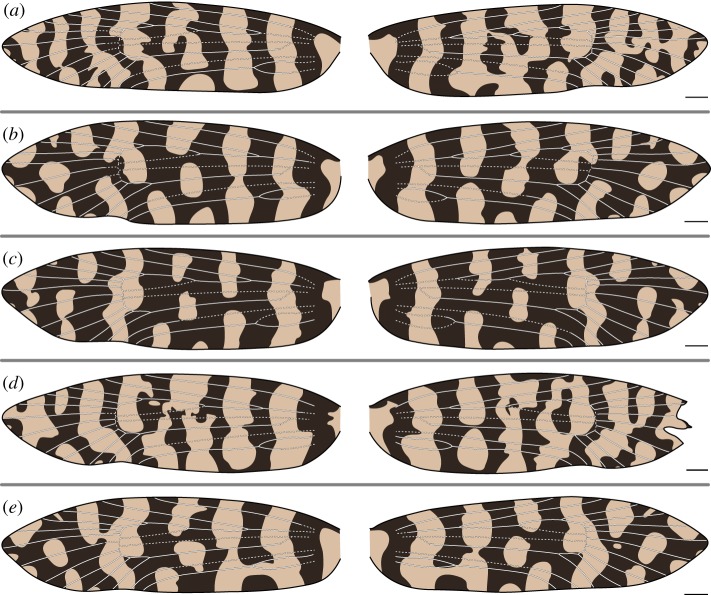
(*a*–*e*) Wing pattern and wing venation of female *Moerarchis australasiella*. All scale bars represent 1 mm.

**Figure 5.**
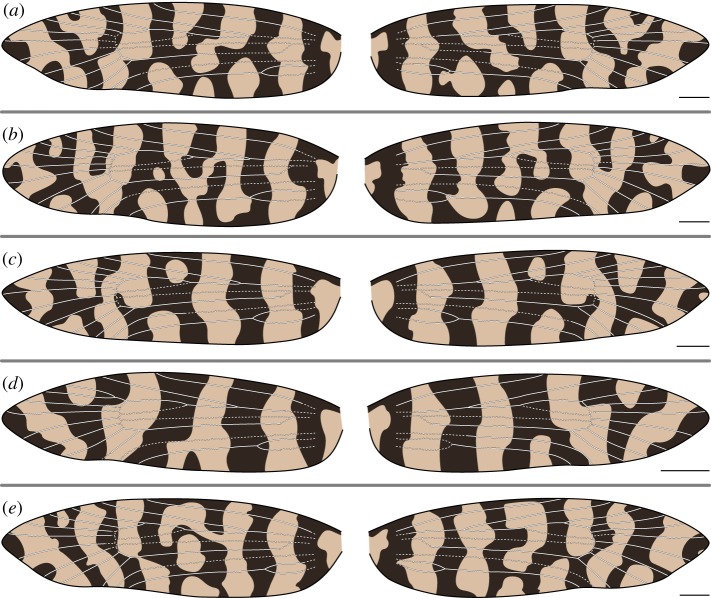
(*a*–*e*) Wing pattern and wing venation of male *Moerarchis australasiella*. All scale bars represent 1 mm.

In *M. australasiella*, Rs_4_ occasionally terminates past the apex (figure 4*e*) and *Rs*_1_+*Rs*_2_ can become fused (figure 5*d*). The relationship between wing pattern and wing venation occasionally follows the ‘uniform wing-margin’ model (figure 4*d*). However, a light pattern element usually surrounds Rs_3_ (figures 4*a*,*b*,*c*,*e* and 5*a*,*d*,*e*). In other specimens, a single light pattern element surrounds Rs_3_ and Rs_4_ at the costa (figure 5*b*), or light pattern elements surround both Rs_1_ and Rs_3_ (figure 5*c*).

**Figure 6.**
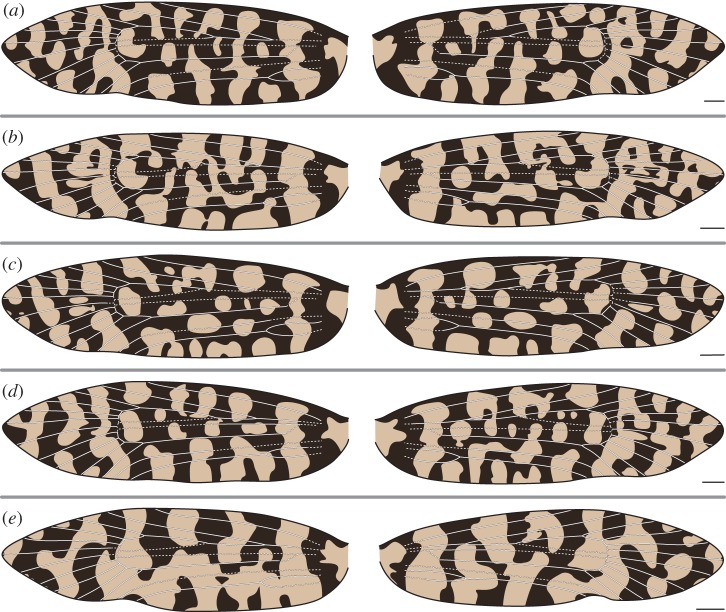
(*a*–*e*) Wing pattern and wing venation of female *Moerarchis clathrata*. All scale bars represent 1 mm.

**Figure 7.**
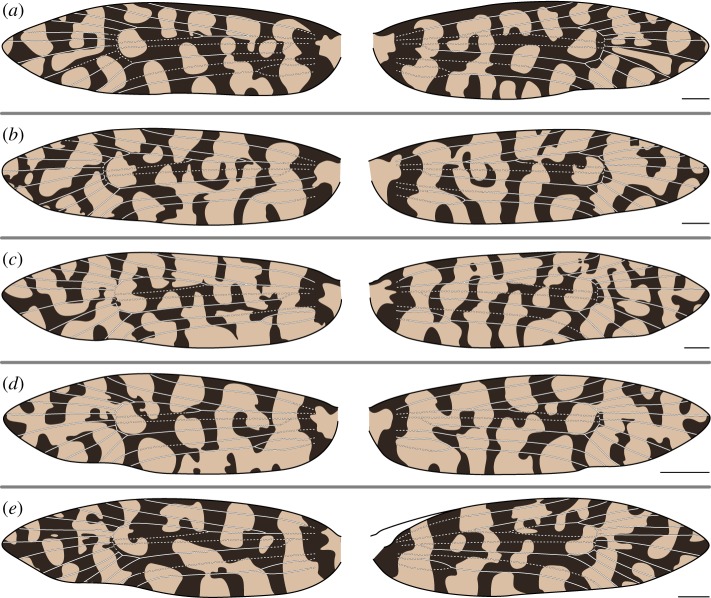
(*a*–*e*) Wing pattern and wing venation of male *Moerarchis clathrata*. All scale bars represent 1 mm.

In both species examined here, CuP is nearly always straddled by a light pattern element. There are only two exceptions: on the right wing of one specimen, CuP is clearly surrounded by dark scales (figure 4*d*), and on the left wing of another specimen, CuP is surrounded by a small patch of dark scales that is only noticeable at high magnification (figure 6*c*). Light wing pattern elements occur between nearly all pairs of adjacent wing veins along the dorsum, as is the case along the costa. In female *M. clathrata*, CuP is the only vein that is ever straddled by a light pattern element. But in males of both *M. clathrata* and *M. australasiella*, *CuA*_1_+*M*_3_ is usually also straddled by a light pattern element (figures 7*a*–*d* and 5*b*–*e*, respectively). A light pattern element also straddles M_3_ in one female *M. australasiella* specimen (figure 4*a*).

Along the costa of some specimens belonging to *M. clathrata*, all veins are surrounded by dark scales and each pair of adjacent veins is separated by a light pattern element, as predicted by the ‘uniform wing-margin’ model (figures 6*a*,*c*,*d* and 7*d*). In one female *M. clathrata* specimen examined, the dark pattern element surrounding Rs_4_ is greatly reduced, such that it appears to be absent if the specimen or illustration is not viewed at high magnification (figure 6*e*), and in another, Rs_3_ is surrounded by a broad light pattern element on only one wing (figure 6*b*). In three of the five male *M. clathrata* specimens examined, Rs_4_ is surrounded by a light pattern element on at least one wing (figure 7*a*–*c*,*e*).

### 3.2. Variation within individuals

A certain amount of variation is always seen between the right and left forewing patterns of the same individual. As noted above, the relationship between pattern and venation can vary slightly, e.g. a vein such as Rs_4_ can be surrounded by light scales on one wing and dark scales on the other (figure 7*b*). In addition, the shape of pattern elements can also vary.

In *M. australasiella*, there is typically little or no variation in the number of pattern elements that reach the wing margin; in one specimen, an apical light pattern element is of small size on the right wing and is absent on the left wing (figure 5*c*). Wing pattern typically varies through the way in which pattern elements become confluent in the interior of the wing, especially in the case of joined bands (figures 4*a*,*d*,*e* and 5*b*,*d*,*e*). Even when the pattern elements of both wings are identical in terms of confluence, the shape of pattern elements varies noticeably (figures 4*b*,*c* and 5*a*).

In *M. clathrata*, the number of pattern elements reaching the wing margin can vary along the costa (figure 6*d*) or, more commonly, along the dorsum (figures 6*b*–*e* and 7). In all specimens examined, there is notable variation between the right and left forewings in the shape of wing pattern elements. However, the two forewings of a single individual are always broadly similar, whether comprised mainly of spots, thin joined bands or wide joined bands.

## 4. Discussion

The results presented here provide insight into transitions between the ‘alternating’ and ‘uniform wing-margin’ models, the effect of vein fusion on wing pattern, the relationship between pattern and venation along the dorsum, the importance of wing size, and the number of bands along the wing. *Moerarchis* demonstrates that highly variable wing patterns can share a common relationship with venation.

### 4.1. Transitions between models

The ‘uniform wing-margin’ model was proposed following observations of the micropterigid species *S. demissa*, *S*. sp. 6 and *S*. sp. 12 [11]. Because the species-level phylogeny for *Sabatinca* is characterized by low support values [23], because wing pattern is highly variable in this genus [11] and because many species remain undescribed and many more probably remain undiscovered [24], the transition from the ‘alternating’ to ‘uniform wing-margin’ models in *Sabatinca* is not understood. *Moerarchis* clarifies one possible mechanism through which this transition can occur.

In all female *M. australasiella* examined here, and in two of the five male specimens, wing pattern always appears to follow the ‘uniform wing-margin’ model from Sc to Rs_2_: each vein is surrounded by dark scales, and light pattern elements reach the costa between each pair of adjacent veins (figures 4 and 5*a*,*b*). In male *M. australasiella*, two specimens lack the light pattern element between Rs_1_ and Rs_2_. In the first specimen, this light pattern element is lacking on both wings, and on one wing, Rs_1_ and Rs_2_ are fused (figure 5*d*). In the second specimen, this light pattern element is present on the left wing but absent on the right wing (figure 5*e*).

Finally, in one male *M. australasiella* specimen, Rs_1_ and Rs_3_ are both straddled by light pattern elements while R, Rs_2_ and Rs_4_ are not (figure 5*c*); the wing pattern follows the ‘uniform wing-margin’ model from the base to the median area, and then follows the ‘alternating wing-margin’ model from the median area to the apex. While this is the only specimen in which a light pattern element straddles Rs_1_, light pattern elements straddle Rs_3_ on both wings of all other male *M. australasiella* specimens (figure 5), on both wings of three female *M. australasiella* specimens (figure 4*b*,*c*,*e*), and on one wing of an additional female specimen (figure 4*a*). In the few remaining specimens of female *M. australasiella*, Rs_3_ is surrounded by a small dark spot that occurs within a wide light band on one wing (figure 4*a*) or on both wings (figure 4*d*).

A similar transition between the two models can be seen on the wings of *M. clathrata*, but Rs_3_ is surrounded by a light pattern element on only one wing of a single specimen (figure 6*b*); Rs_4_ is straddled by a light pattern element (figure 7*a*–*c*,*e*) or nearly abutted by a light pattern element (figure 7*d*) in all specimens examined. The areas of the wing that follow the ‘alternating wing-margin’ model in *M. australasiella* and *M. clathrata* do so in opposite ways, in the sense that Rs_1_ and Rs_3_ can be surrounded by light pattern elements in *M. australasiella*, whereas Rs_4_ is far more commonly surrounded by a light pattern element in *M. clathrata*; in the region surrounding Rs_3_ and Rs_4_, what is light in *M. australasiella* is dark in *M. clathrata* and vice versa. This ‘flipping’ of light and dark pattern elements between species has also been found in Micropterigidae [11] and Psychidae [16].

**Figure 8.**
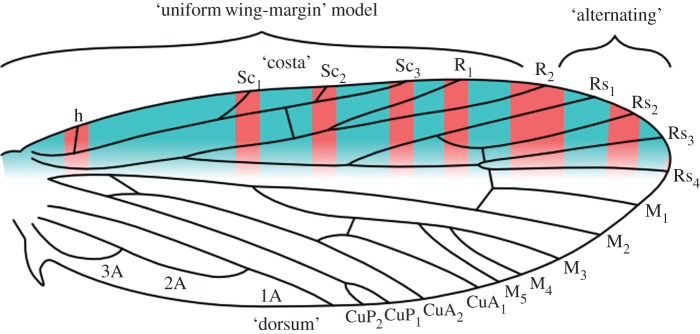
A hypothetical schematic showing a transition between the ‘uniform’ and ‘alternating wing margin’ models, based on figure 5*c*.

The generalization can be made that *Moerarchis* wing patterns tend to follow the ‘uniform wing-margin’ model toward the base of the wing and the ‘alternating wing-margin’ model towards the apex, with the boundary between these two models shifting within the region where the Rs veins terminate along the costa. Because different pattern elements on the very same wing can follow both variants of the ‘wing-margin’ model, these two variants should be conceptualized as endpoints along a single continuum rather than mutually exclusive character states for entire wings or species (figure 8).

### 4.2. Fusion of veins

Various plesiomorphic lepidopteran wing veins appear to be ‘lost’ in derived lineages. This can occur via one of two mechanisms: fusion—whereby two veins continue to be expressed in the adult wing, but cannot be visually distinguished from each other—or true lack of expression. Because partial fusion of veins has been observed in countless lineages, and because veins that appear to be ‘lost’ are occasionally regained [25–28], fusion may be the most parsimonious explanation for vein ‘loss’ [14]. However, the true prevalence of fusion versus lack of expression is unknown, as is the effect of vein fusion on wing pattern.

In a specimen examined here, Rs_1_ and Rs_2_ are partially fused on the left wing and are completely fused on the right wing, with the fused vein terminating along the costa at the position associated with Rs_2_ (figure 5*d*). Rs_1_, therefore, appears to be ‘lost’ on this wing. The two wings have identical patterns along the costa despite the difference in venation. This observation is consistent with previous findings which have indicated that the ancestral location of wing veins determines pattern development, regardless of whether veins are present in a differentiated form in the adult wing [10,11]. The ‘loss’ of a vein via fusion does not affect pattern development in *Moerarchis*.

### 4.3. The dorsal wing margin

At present, both variants of the ‘wing-margin’ model have been evaluated only for the costal margin of the wing. This model was originally based on Olethreutinae and is considered to apply to all Tortricidae [12]; moths in this family have up to five bands that run from the costa to the dorsum, but venation is so reduced that few veins reach the dorsum [13]. In Micropterigidae, which have a relatively complete suite of plesiomorphic lepidopteran veins, confluence of pattern elements along the dorsum obscures any possible relationship between venation and pattern along this margin of the wing [10,11].

But in *Moerarchis*, wing pattern elements are as finely differentiated along the dorsum as they are along the costa. Along the dorsum, the relationship between pattern and venation is similar to that along the costa, as follows. The ‘uniform wing margin’ model usually holds, with veins typically surrounded by dark scales, and light pattern elements usually occurring between each pair of adjacent veins. One vein is typically straddled by a light pattern element: Rs_3_ in *M. australasiella* and Rs_4_ in *M. clathrata*, along the costa; CuP along the dorsum. And one additional vein is sometimes straddled by a light pattern element: Rs_1_ along the costa in *M. australasiella* (figure 5*c*), and M_3_ along the dorsum (figures 4*a*, 5, 7*a*–*d*).

The same veins are straddled by light pattern elements along the dorsum of both species—M_3_ and CuP—but different veins are straddled by light pattern elements along the costa in the two species—Rs_3_ in *M. australasiella* and Rs_4_ in *M. clathrata*. This suggests that the relationship between wing pattern and wing venation along the costa is decoupled from that along the dorsum, though far more data are needed to fully address this issue.

*Moerarchis* is generally considered to have typical tineid wing venation [19], though one vein branch is not visible on the adult wing of males belonging to *M. australasiella* and *M. clathrata*. One branch of the CuA vein is considered to have been lost in many tineid genera, but these are all narrow-winged [19]. In two genera, *Monopis* and *Setomorpha*, M_3_ and CuA_1_ are stalked, or partially fused [19]. Here it is assumed that M_3_ and CuA_1_ are fused in the males of *Moerarchis*; this assumption is very tenuous, and does not have a major impact on the interpretation of wing patterns.

Unknown vein homologies are another unresolved issue. A four-branched M vein has been known from *Agathiphaga queenslandensis* for decades [29]. A more recent examination of *Agathiphaga vitiensis* from Vanuatu found up to 10 M, Cu and A veins reaching the wing margin: 5 M veins, 2 CuA veins, 2 CuP veins and a fused A vein [14]. A branched CuP vein and a fourth or fifth branch of the M vein are unknown outside of *Agathiphaga* [14], such that the wing venation ground plan for Lepidoptera is usually reconstructed with a three-branched M vein and an unbranched CuP vein [30]. The three M veins visible on the wings of *Moerarchis* are referred to here as M_1_, M_2_ and M_3_; this terminology is strictly positional as homologies remain unknown between the three M veins in *Moerarchis*—and all Lepidoptera other than *Agathiphaga*—and the five M veins in *A. vitiensis*. Because plesiomorphic wing veins continue to influence the development of wing pattern even when not expressed in the adult wing [10,11,14], homologies between M veins must be understood in order for findings from taxa such *Moerarchis* to be placed in broader evolutionary context.

### 4.4. Wing size

In general, smaller, microlepidopteran moths are considered to have simpler wing patterns than larger moths such as the Papilionoidea, Bombycoidea and Geometridae [31,32]. This observation might be stem from the fact that larger wings have more space for complex patterns, or it could be owing to advanced developmental mechanisms that originated within certain derived lineages of moths. The intricacy of wing patterns in the Hepialidae [6], a very early-diverged, but large-bodied, family belonging to the Homoneura [33], demonstrates that complex wing patterns can originate in early-diverged moth lineages, given sufficient wing surface area. However, the relative contributions of phylogeny and wing size to wing pattern complexity have not been tested explicitly. This question is beyond the scope of the present contribution; however, *Moerarchis* provides preliminary intraspecific data that can inform future studies.

In *M. clathrata*, the number of differentiated pattern elements—or classification into the ‘joined’ or ‘spotted’ categories—can be deceiving in that these criteria do not necessarily correspond with the number of discrete light and dark patches of colour that reach the wing margin. In *M. australasiella*, however, the number of discrete pattern elements does correspond to the number of light or dark patches of colour that reach the wing margin, making the characterization of wing pattern far more straightforward. The significant correlation between wing size and number of pattern elements in *M. australasiella* shows that wing size can underlie wing pattern complexity in the absence of any phylogenetic influence. Of course, number of pattern elements is just one measure of wing pattern complexity. In other moth lineages, additional measures of wing pattern complexity could include the number of colours, the number of types of pattern elements—bands, ripple patterns, etc.—or the complexity of individual pattern elements such as symmetry systems.

### 4.5. Patterns with more than six bands

Some moths have wing patterns with more than six transverse bands. Lemche’s ‘vein-fork’ model for the relationship between wing pattern and wing venation predicts a primitive ground plan with seven dark bands [9], but no empirical support has been found for his predictive model, and recent examinations of Micropterigidae have recovered a primitive wing pattern ground plan for Lepidoptera with only five or six bands [10,11].

The ‘uniform wing-margin’ model was originally proposed based on *Sabatinca* species with small spots surrounding each vein at the costa [11]; no wing pattern had yet been shown to consist of transverse bands running from the dorsum to the costa, with each band surrounding only one vein at the costa. Various Australian psychids have wing patterns with transverse bands that follow the ‘uniform wing-margin’ model, but fewer than seven bands are present [16]. Certain specimens of *M. australasiella* figured here have more than six dark bands on the wing; when viewed simultaneously with wing venation, one such specimen provides, to my knowledge, the first empirical confirmation of the ‘uniform wing-margin’ model with more than six bands (figure 4*a*).

### 4.6. Future directions

In general, more data are needed in order to determine the relevance of the two ‘wing-margin’ models to various lineages of microlepidoptera; additional studies would allow the analysis of wing pattern in a robust phylogenetic context. This particular study raises two specific issues that should be addressed.

The first issue regards transitions between the ‘alternating’ and ‘uniform wing-margin’ models. The frequency with which a transition can be seen on a single wing is completely unknown outside of *Moerarchis*. Furthermore, constraints on the directionality of such transitions remain unknown: it is possible, but certainly not confirmed, that wing patterns could follow the ‘alternating wing-margin’ model at the base of the wing, and the ‘uniform wing-margin’ model towards the apex.

The second issue regards wing pattern elements that extend from the costal to the dorsal margin. The present study has found that wing pattern elements can have a uniform relationship with venation along the entirety of the wing margin. Wing pattern in *Moerarchis* could be described as consisting of individual spots that are associated with only one margin of the wing, which can become confluent with any number of spots along the opposite margin. In other words, in *Moerarchis* many pattern elements have inconsistent relationships with the costal versus dorsal wing margins. This may not have been the case in ancestral moths, but plesiomorphic wing pattern along the dorsum remains unknown because of the high degree of confluence of pattern elements along the dorsum in the Micropterigidae [10,11]. Now that a consistent venation-pattern relationship has been established along the entirety of the wing margin in certain microlepidoptera, the next step is to determine whether bands of colour on the wings of ancestral moths may have had consistent relationships with particular veins along both the costa and the dorsum.

## 5. Conclusion

The wings of *Moerarchis* figured here address various long-standing questions regarding the evolution and development of microlepidopteran wing pattern. Firstly, *Moerarchis* demonstrates that a transition can occur between the ‘uniform’ and ‘alternating wing-margin’ models on a single wing: pattern can follow one model towards the base of the wing, and another towards the apex. Second, *Moerarchis* demonstrates that fusion of veins does not affect the development of wing pattern; when veins fuse, pattern elements remain in alignment with the plesiomorphic locations of wing veins. Third, *Moerarchis* demonstrates that the relationship between venation and pattern along the costal wing margin can also be observed along the dorsal margin. Fourth, intraspecific variation in wing size is shown to influence the number of discrete pattern elements that occur on the wings of *M. australasiella*. And lastly, *M. australasiella* provides strong empirical support for previous speculation that wing patterns with more than six dark bands can develop in accordance with the ‘uniform wing-margin’ model.

## Supplementary Material

Supplementary Figures and Tables

## Data accessibility

This study is based on observations of insect wings, all of which are figured in either the main body or the electronic supplementary material of this paper.
