# Supplementary material for: The wing pattern of Moerarchis Durrant, 1914 (Lepidoptera: Tineidae) clarifies transitions between predictive models
Source: R Soc Open Sci. 2017 Mar 1;4(3):161002. doi: 10.1098/rsos.161002 (PMC5383847; doi:10.1098/rsos.161002)
Supplement: Supplementary Figures and Tables [file rsos161002supp1.pdf]

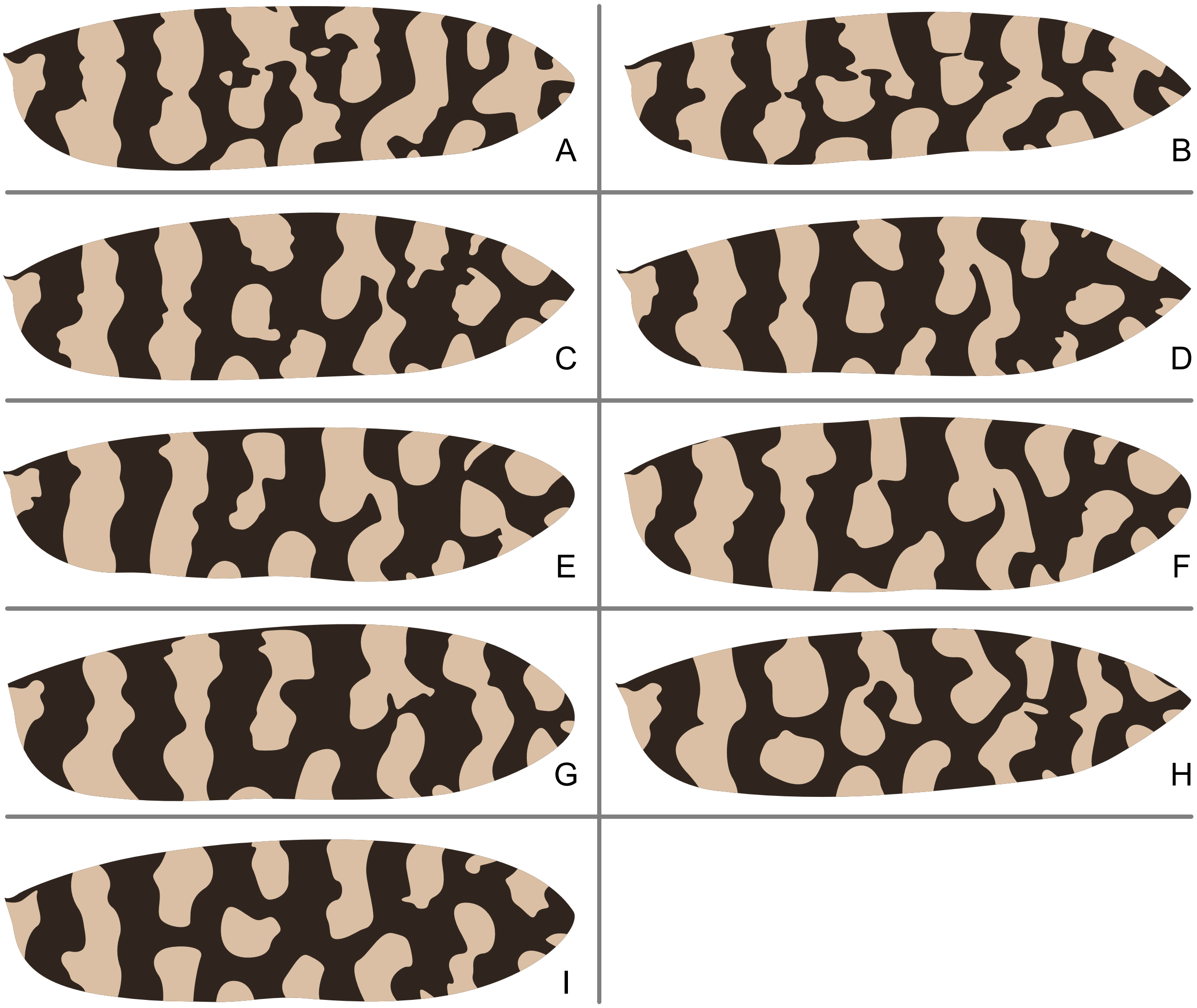

Figure S1: Wing pattern of female *Moerarchis australasiella*. Specimen numbers: A S154 (used in Mann-Whitney-Wilcoxon Test: “many” group), B S157 (used in Mann-Whitney-Wilcoxon Test: “many” group), C S151 (used in Mann-Whitney-Wilcoxon Test: “many” group), D S163 (used in Mann-Whitney-Wilcoxon Test: “many” group), E S161 (used in Mann-Whitney-Wilcoxon Test: “many” group), F S138 (used in Mann-Whitney-Wilcoxon Test: “many” group), G S149 (used in Mann-Whitney-Wilcoxon Test: “many” group), H S141, I S171 (used in Mann-Whitney-Wilcoxon Test: “many” group).

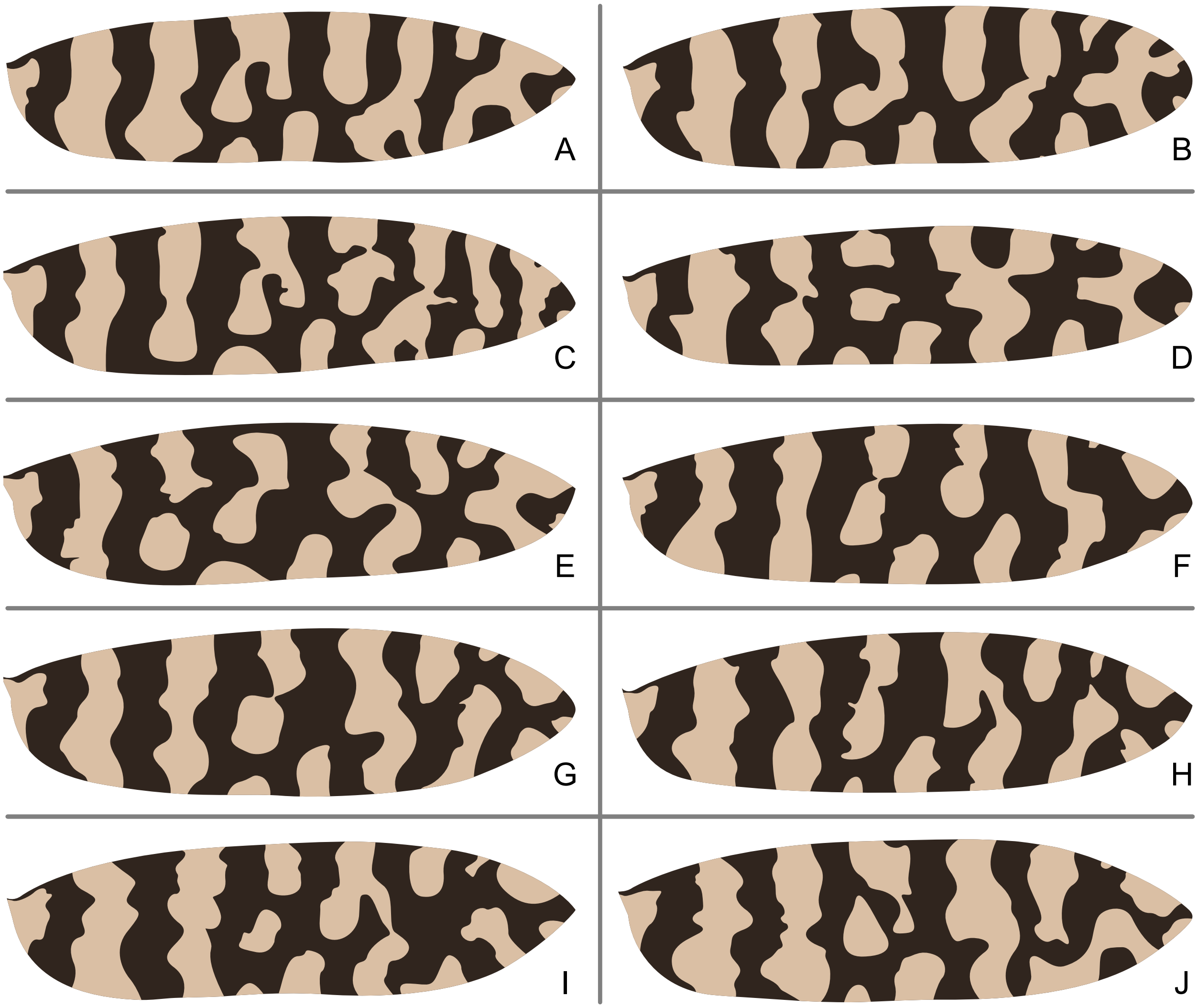

Figure S2: Wing pattern of female *Moerarchis australasiella*. Specimen numbers: A S160 (used in Mann-Whitney-Wilcoxon Test: "many" group), B S150 (used in Mann-Whitney-Wilcoxon Test: "many" group), C S142, D S158, E S166, F S170, G S155, H S190, I S156, J S148.

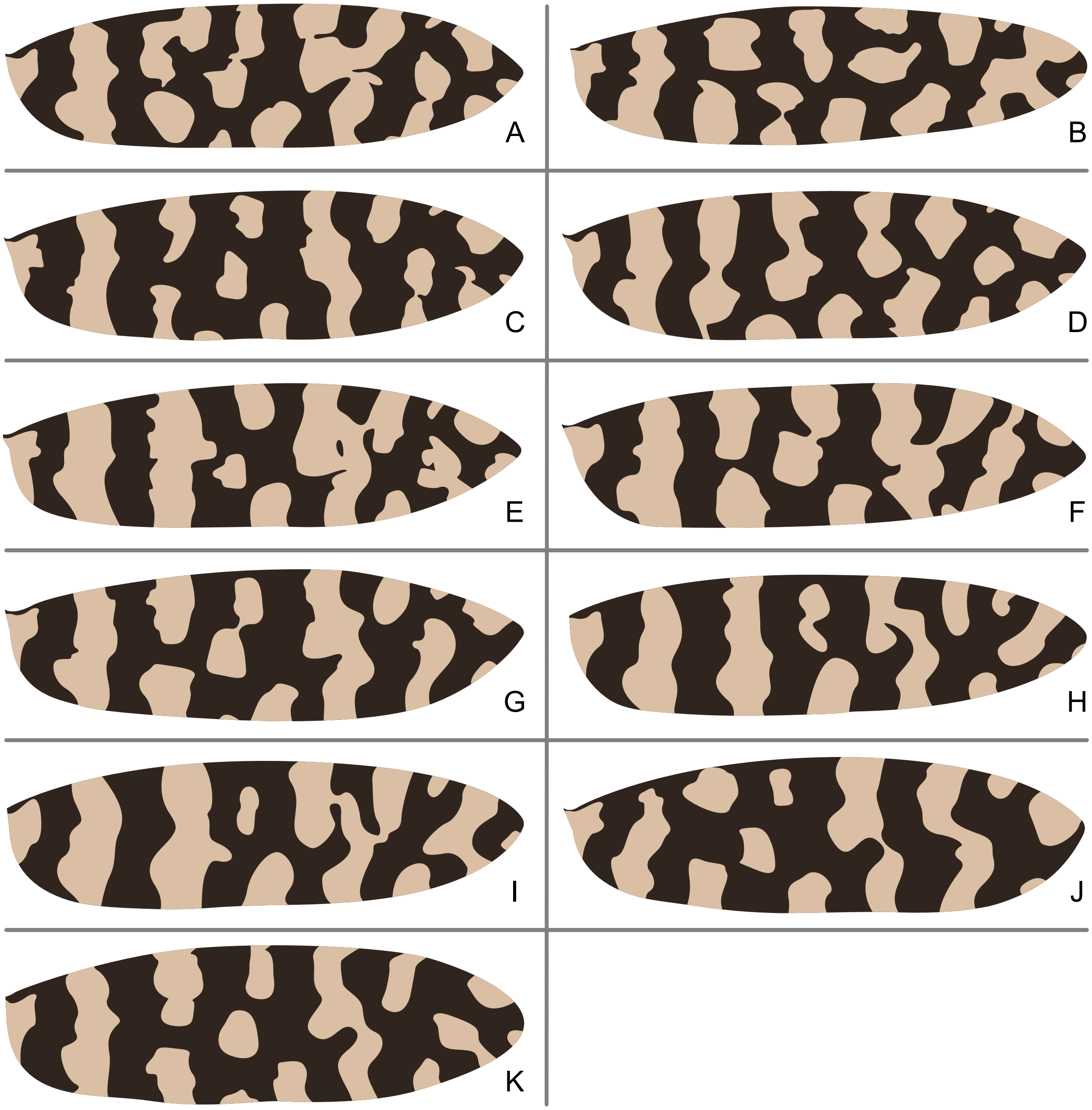

Figure S3: Wing pattern of female *Moerarchis australasiella*. Specimen numbers: A S180, B S159, C S152, D S167, E S164, F S143, G S189, H S177, I S186, J S139, K S146.

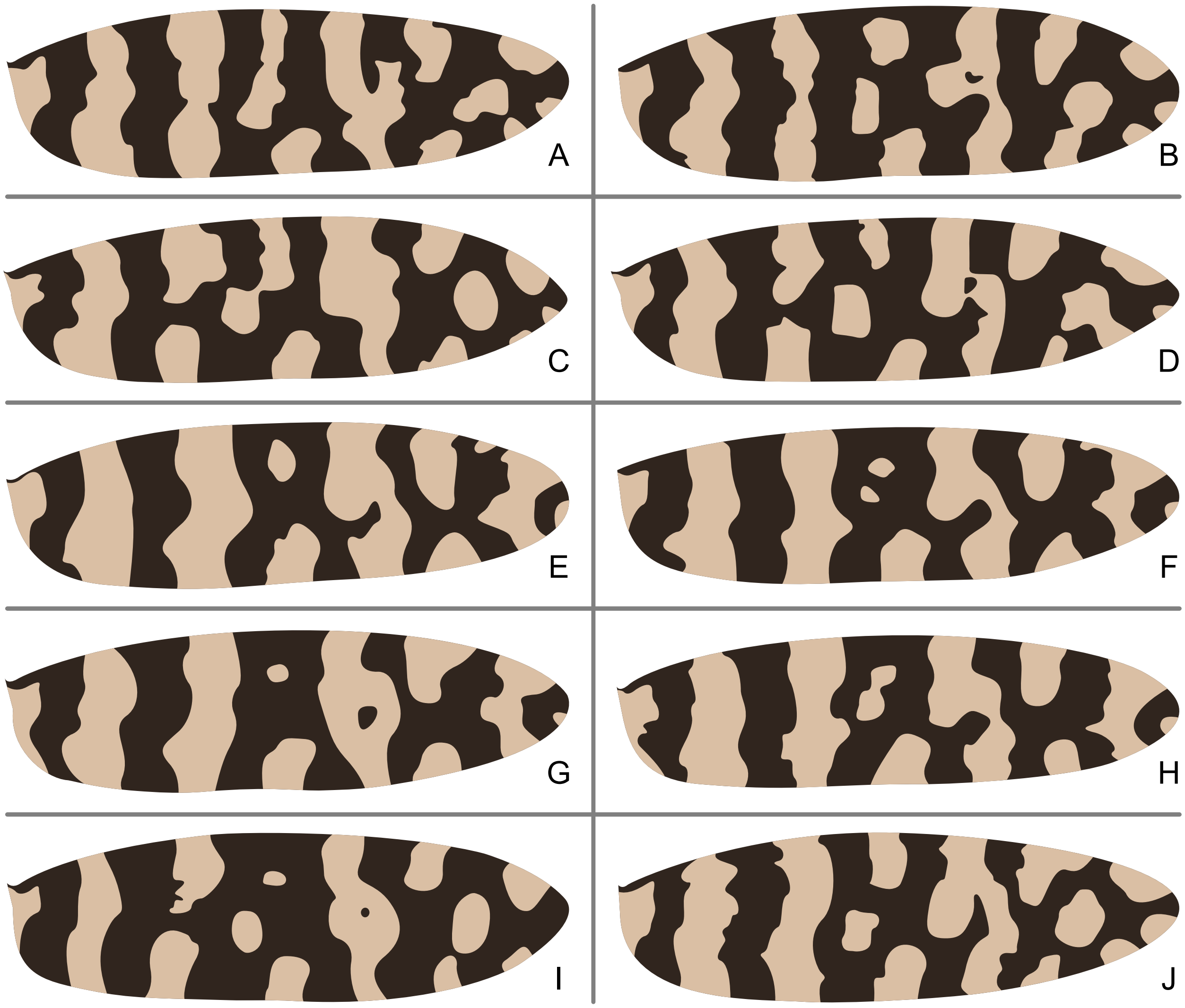

Figure S4: Wing pattern of female *Moerarchis australasiella*. Specimen numbers: A S153, B S140, C S169, D S162, E S179, F S182, G S175, H S173, I S176, J S165.

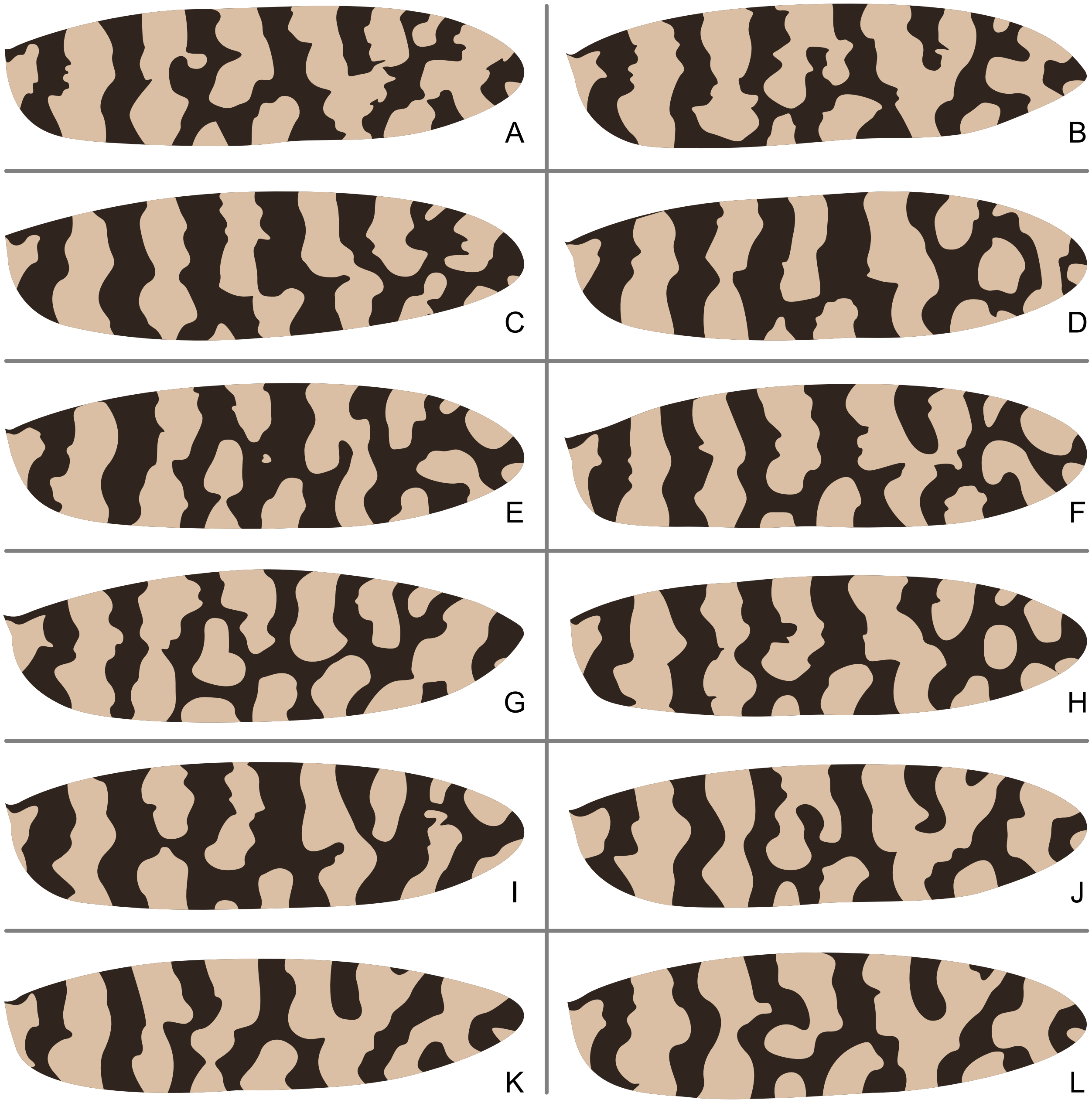

Figure S5: Wing pattern of male *Moerarchis australasiella*. Specimen numbers: A S074 (used in Mann-Whitney-Wilcoxon Test: “many” group), B S099 (used in Mann-Whitney-Wilcoxon Test: “many” group), C S070 (used in Mann-Whitney-Wilcoxon Test: “many” group), D S063 (used in Mann-Whitney-Wilcoxon Test: “many” group), E S072, F S086 (used in Mann-Whitney-Wilcoxon Test: “many” group), G S065, H S109 (used in Mann-Whitney-Wilcoxon Test: “many” group), I S113 (used in Mann-Whitney-Wilcoxon Test: “many” group), J S077 (used in Mann-Whitney-Wilcoxon Test: “many” group), K S079 (used in Mann-Whitney-Wilcoxon Test: “many” group), L S137 (used in Mann-Whitney-Wilcoxon Test: “many” group).

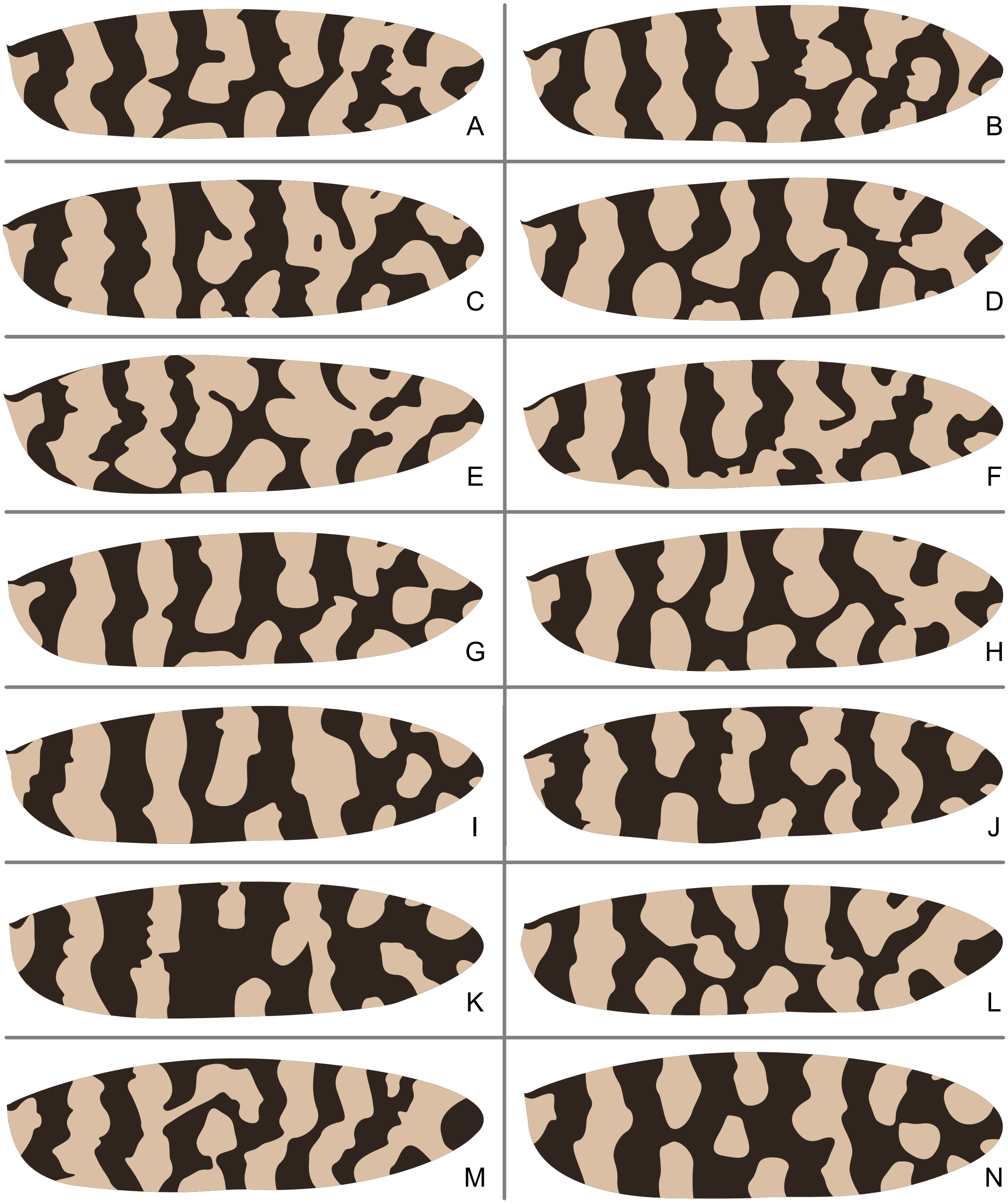

Figure S6: Wing pattern of male *Moerarchis australasiella*. Specimen numbers: A S118, B S092, C S105, D S098, E S108, F S075, G S104, H S073, I S135, J S064, K S121, L S081, M S136, N S069.

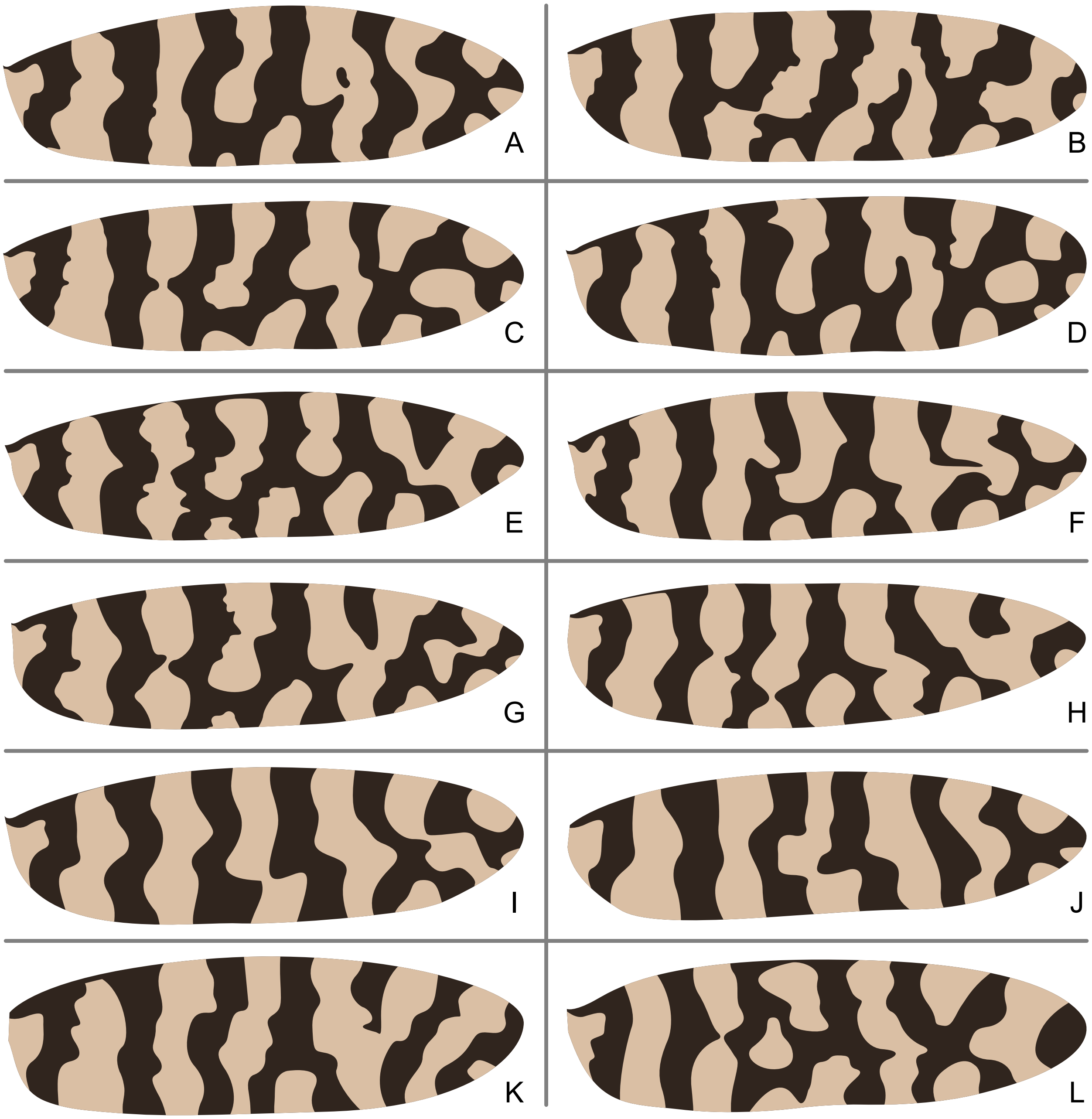

Figure S7: Wing pattern of male *Moerarchis australasiella*. Specimen numbers: A S101, B S088, C S089, D S096, E S083, F S111, G S106, H S117, I S076, J S122, K S114, L S094.

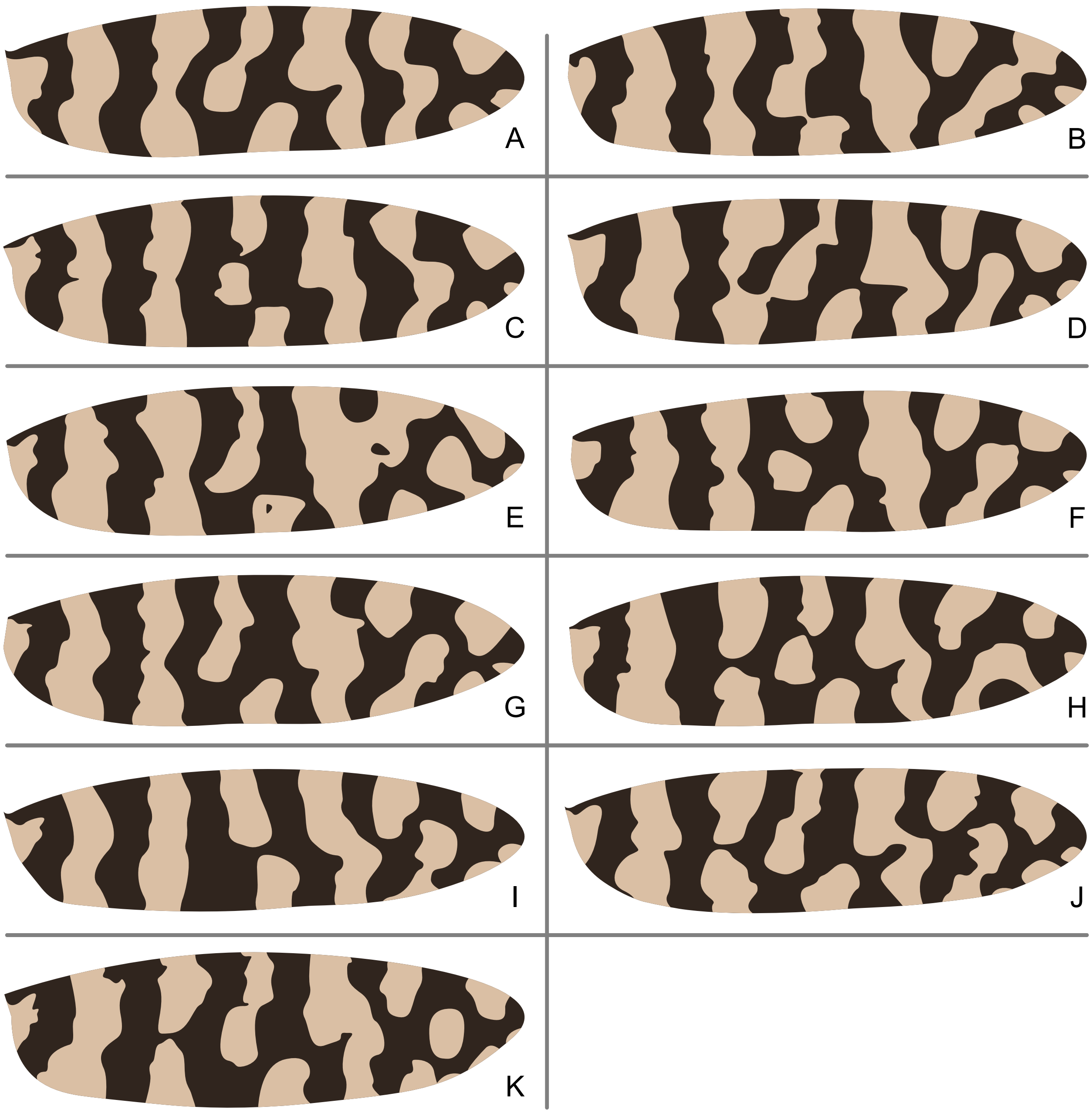

Figure S8: Wing pattern of male *Moerarchis australasiella*. Specimen numbers: A S102, B S095, C S091, D S080, E S128, F S112, G S097, H S078, I S067, J S100, K S090.

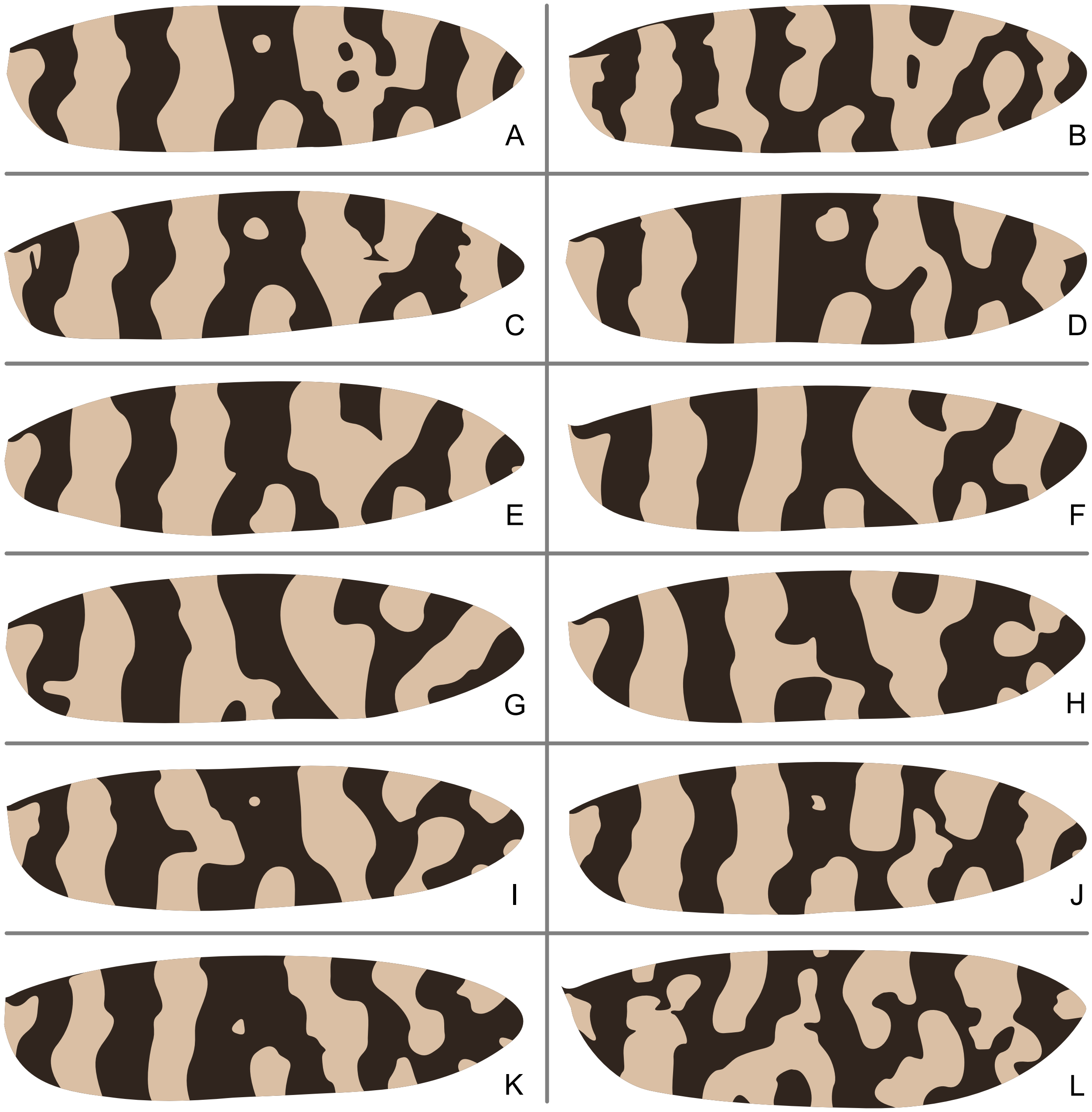

Figure S9: Wing pattern of male *Moerarchis australasiella*. Specimen numbers: A S120 (used in Mann-Whitney-Wilcoxon Test: “few” group), B S093, C S123 (used in Mann-Whitney-Wilcoxon Test: “few” group), D S119 (used in Mann-Whitney-Wilcoxon Test: “few” group), E S124 (used in Mann-Whitney-Wilcoxon Test: “few” group), F S126 (used in Mann-Whitney-Wilcoxon Test: “few” group), G S127 (used in Mann-Whitney-Wilcoxon Test: “few” group), H S110 (used in Mann-Whitney-Wilcoxon Test: “few” group), I S130 (used in Mann-Whitney-Wilcoxon Test: “few” group), J S133 (used in Mann-Whitney-Wilcoxon Test: “few” group), K S132 (used in Mann-Whitney-Wilcoxon Test: “few” group), L S071.

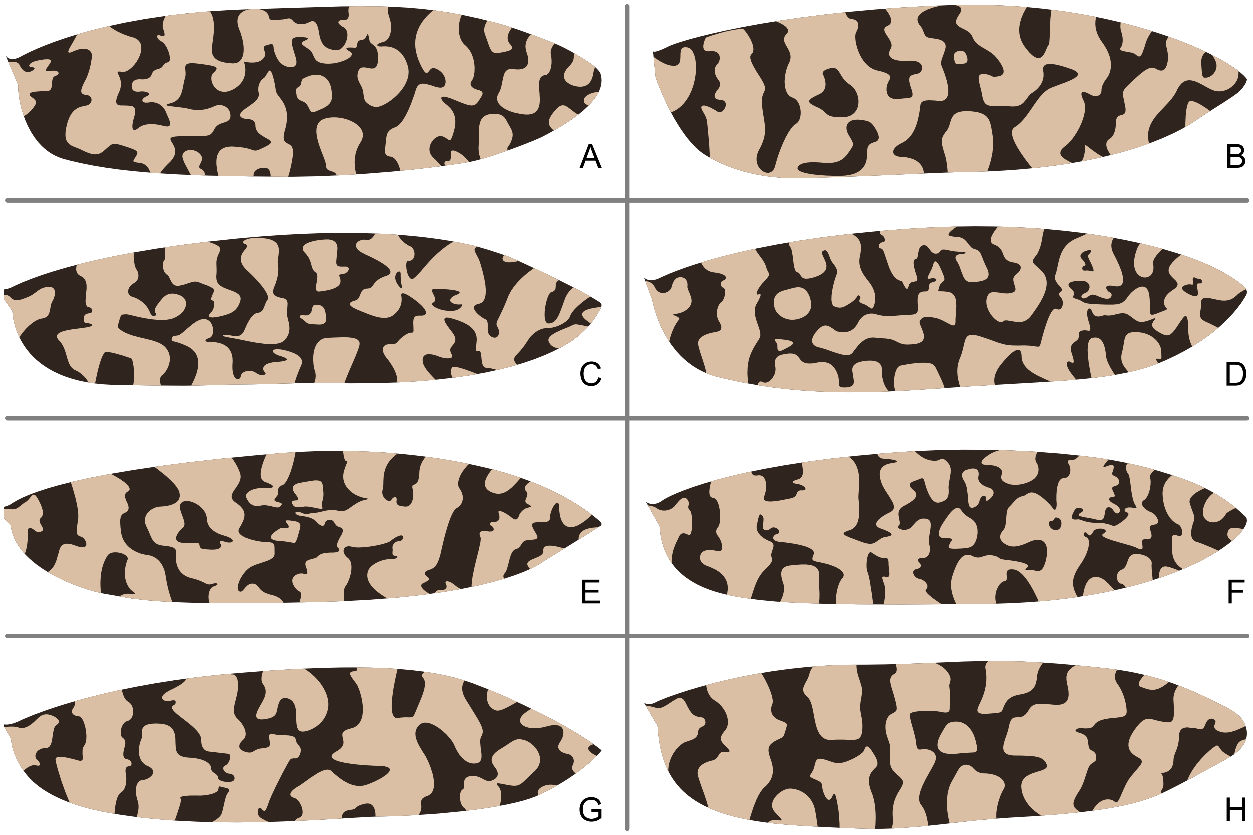

Figure S10: Wing pattern of female *Moerarchis clathrata*. Specimen numbers: A S034 (used in Mann-Whitney-Wilcoxon Test: “joined” group), B S060, C S056 (used in Mann-Whitney-Wilcoxon Test: “joined” group), D S038 (used in Mann-Whitney-Wilcoxon Test: “joined” group), E S058 (used in Mann-Whitney-Wilcoxon Test: “joined” group), F S055 (used in Mann-Whitney-Wilcoxon Test: “joined” group), G S062, H S051 (used in Mann-Whitney-Wilcoxon Test: “joined” group).

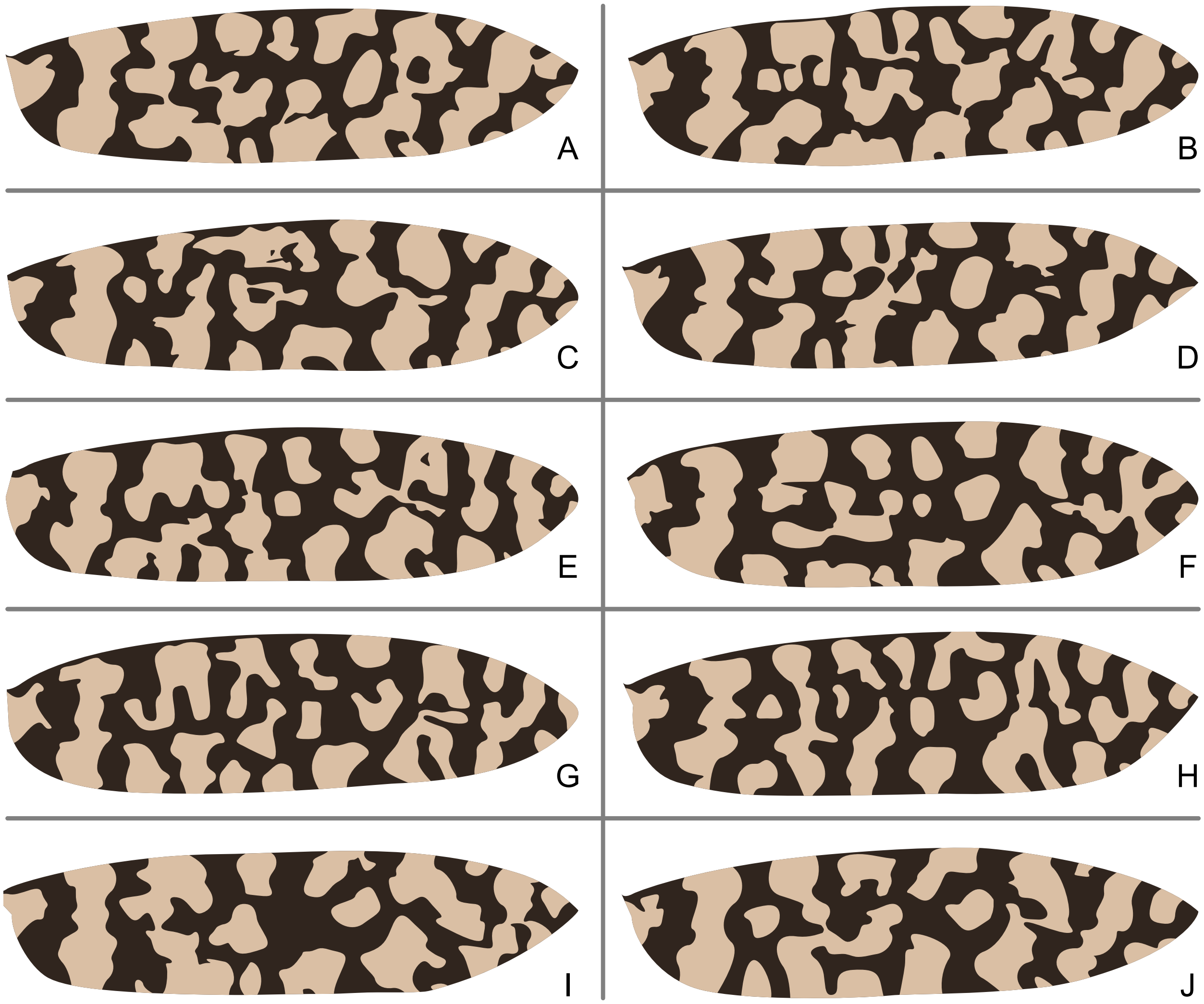

Figure S11: Wing pattern of female *Moerarchis clathrata*. Specimen numbers: A S039 (used in Mann-Whitney-Wilcoxon Test: "joined" group), B S033 (used in Mann-Whitney-Wilcoxon Test: "joined" group), C S046, D S040 (used in Mann-Whitney-Wilcoxon Test: "joined" group), E S037 (used in Mann-Whitney-Wilcoxon Test: "joined" group), F S049, G S053 (used in Mann-Whitney-Wilcoxon Test: "spotted" group), H S035 (used in Mann-Whitney-Wilcoxon Test: "spotted" group), I S044, J S043.

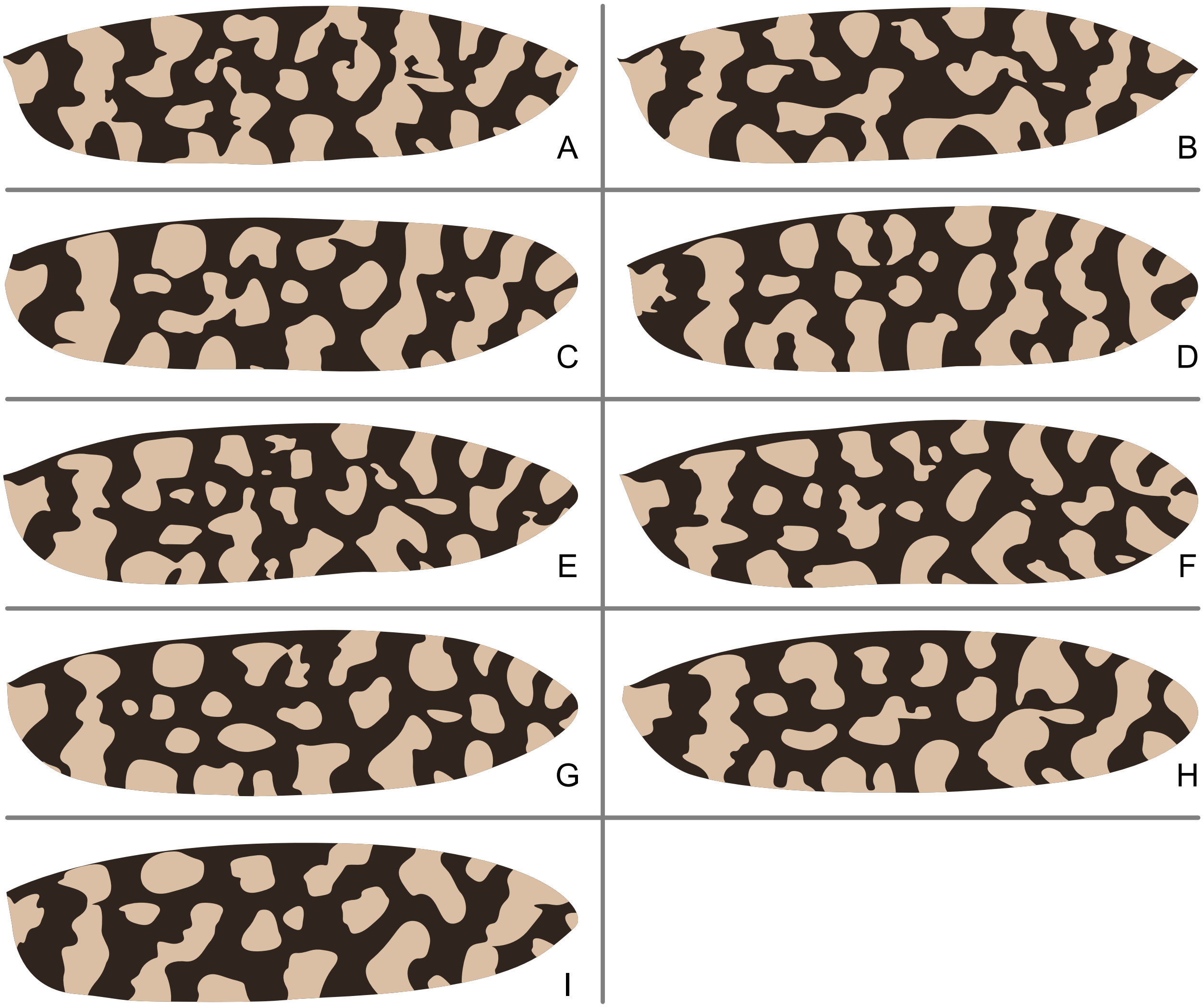

Figure S12: Wing pattern of female *Moerarchis clathrata*. Specimen numbers: A S036 (used in Mann-Whitney-Wilcoxon Test: “spotted” group), B S054 (used in Mann-Whitney-Wilcoxon Test: “spotted” group), C S042 (used in Mann-Whitney-Wilcoxon Test: “spotted” group), D S059 (used in Mann-Whitney-Wilcoxon Test: “spotted” group), E S048 (used in Mann-Whitney-Wilcoxon Test: “spotted” group), F S057 (used in Mann-Whitney-Wilcoxon Test: “spotted” group), G S041 (used in Mann-Whitney-Wilcoxon Test: “spotted” group), H S047 (used in Mann-Whitney-Wilcoxon Test: “spotted” group), I S045.

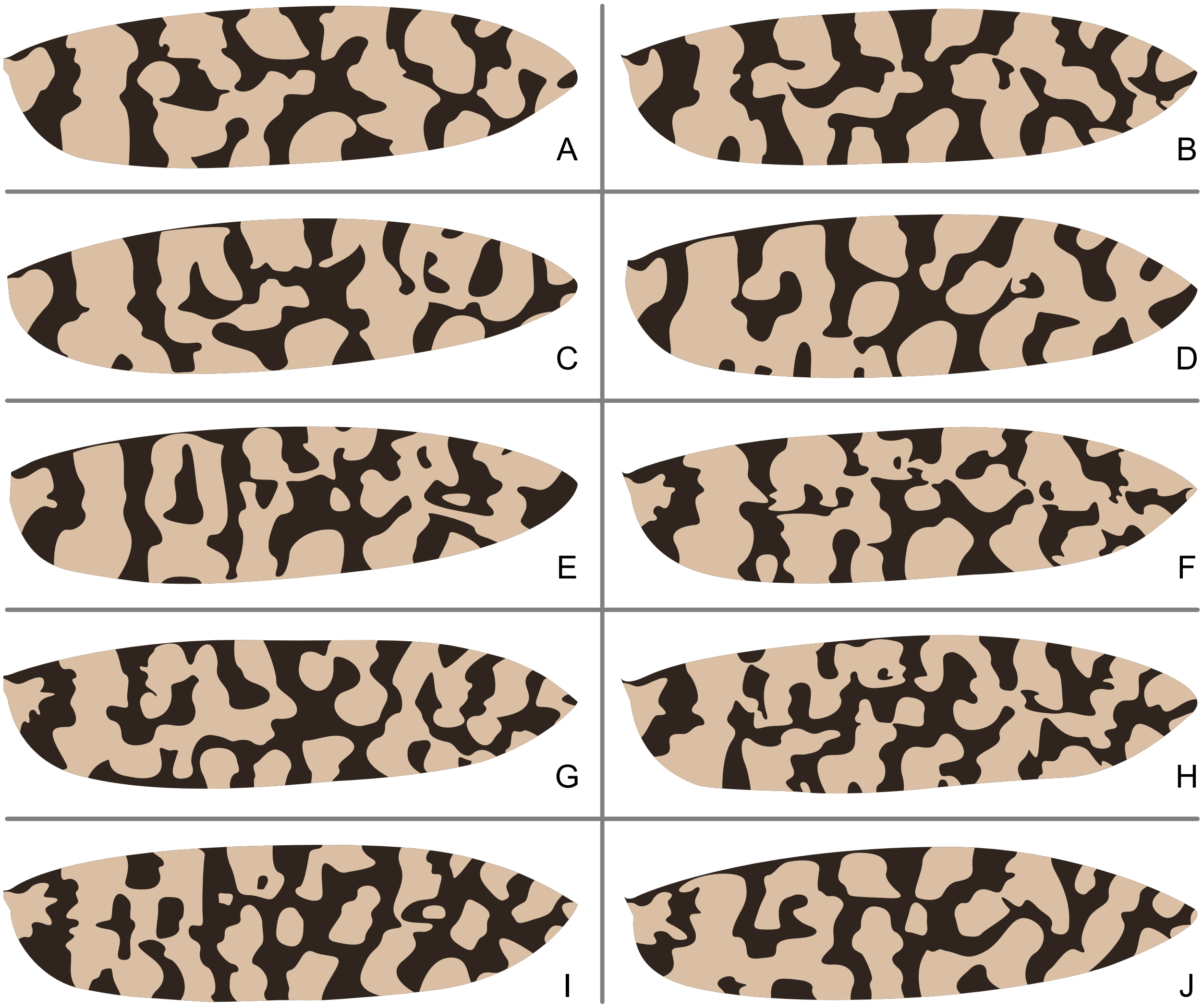

Figure S13: Wing pattern of male *Moerarchis clathrata*. Specimen numbers: A S032, B S015 (used in Mann-Whitney-Wilcoxon Test: "joined" group), C S016 (used in Mann-Whitney-Wilcoxon Test: "joined" group), D S018 (used in Mann-Whitney-Wilcoxon Test: "joined" group), E S011 (used in Mann-Whitney-Wilcoxon Test: "joined" group), F S005 (used in Mann-Whitney-Wilcoxon Test: "joined" group), G S004 (used in Mann-Whitney-Wilcoxon Test: "joined" group), H S017 (used in Mann-Whitney-Wilcoxon Test: "joined" group), I S009 (used in Mann-Whitney-Wilcoxon Test: "joined" group), J S002 (used in Mann-Whitney-Wilcoxon Test: "joined" group).

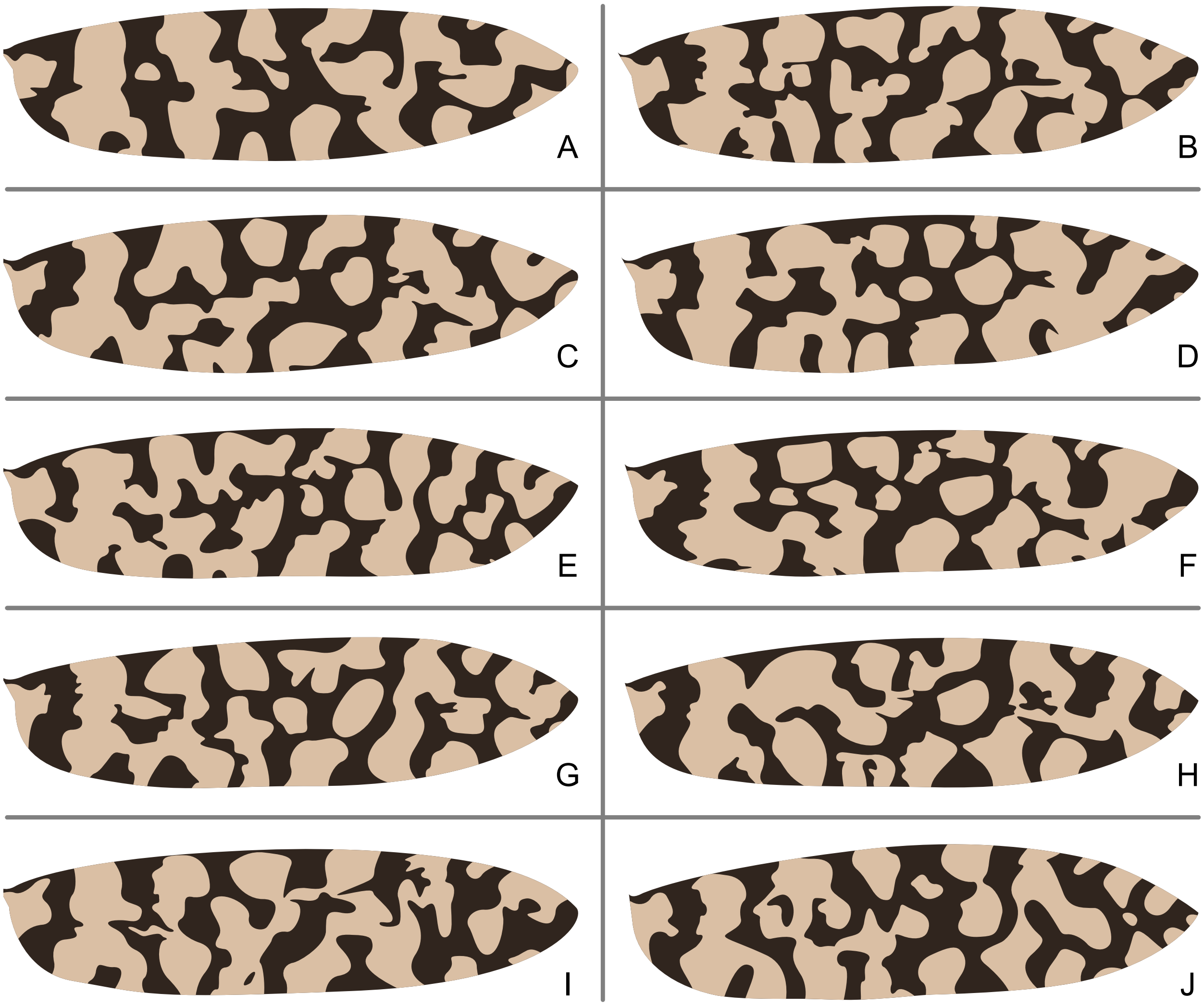

Figure S14: Wing pattern of male *Moerarchis clathrata*. Specimen numbers: A S019, B S020, C S012, D S028 (used in Mann-Whitney-Wilcoxon Test: “joined” group), E S010, F S029, G S030, H S001, I S014, J S008.

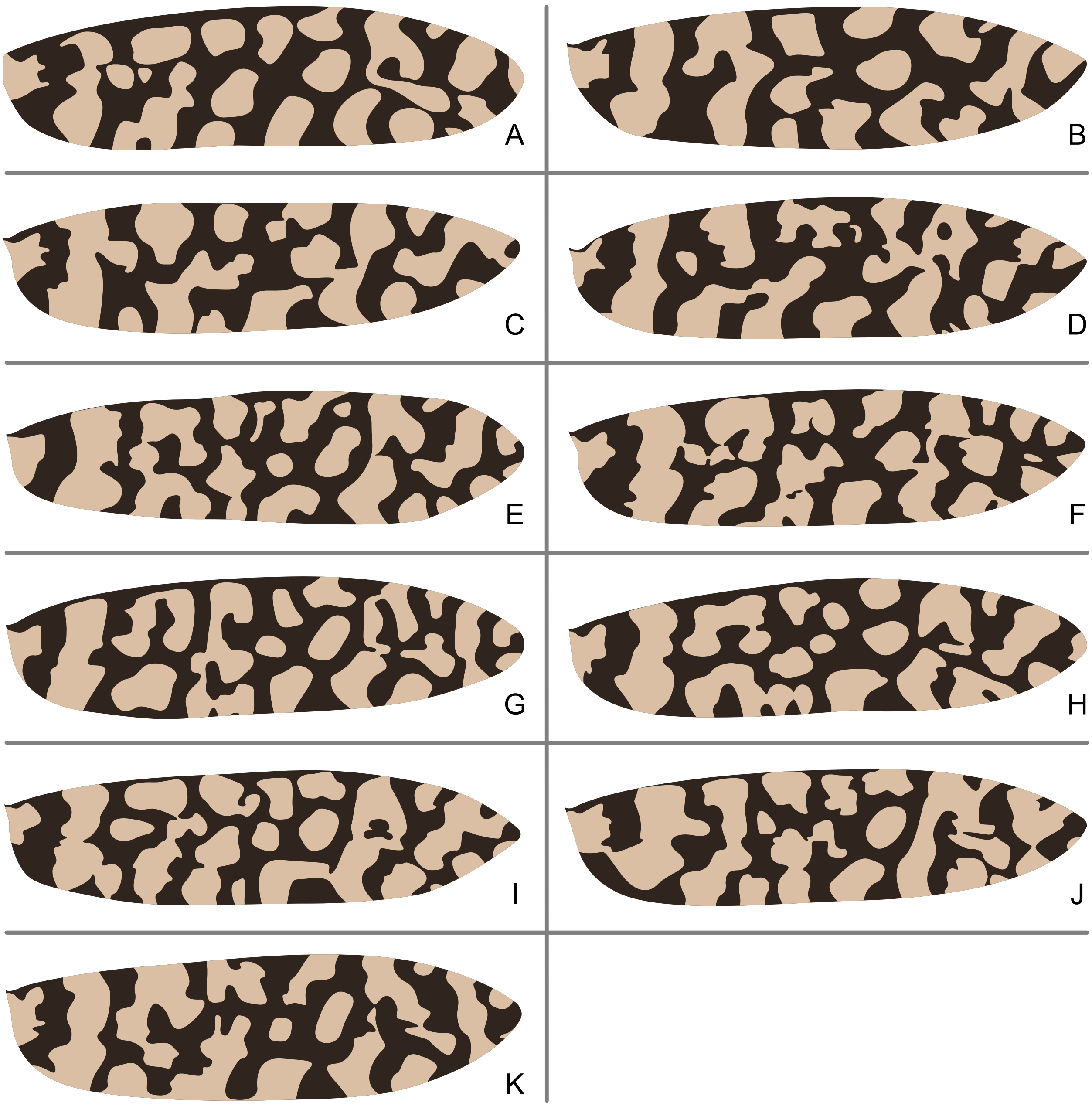

Figure S15: Wing pattern of male *Moerarchis clathrata*. Specimen numbers: A S006 (used in Mann-Whitney-Wilcoxon Test: “spotted” group), B S022 (used in Mann-Whitney-Wilcoxon Test: “spotted” group), C S013, D S025 (used in Mann-Whitney-Wilcoxon Test: “spotted” group), E S024 (used in Mann-Whitney-Wilcoxon Test: “spotted” group), F S026 (used in Mann-Whitney-Wilcoxon Test: “spotted” group), G S031 (used in Mann-Whitney-Wilcoxon Test: “spotted” group), H S027 (used in Mann-Whitney-Wilcoxon Test: “spotted” group), I S021 (used in Mann-Whitney-Wilcoxon Test: “spotted” group), J S023 (used in Mann-Whitney-Wilcoxon Test: “spotted” group), K S003 (used in Mann-Whitney-Wilcoxon Test: “spotted” group).

Species	Sex	Category	Specimen numbers
M. australasiella	F	“few”	S140, S153, S162, S165, S169, S173, S175, S176, S179, S182
M. australasiella	F	“many”	S138, S149, S150, S151, S154, S157, S160, S161, S163, S171
M. australasiella	M	“few”	S110, S119, S120, S123, S124, S126, S127, S130, S132, S133
M. australasiella	M	“many”	S063, S070, S074, S077, S079, S086, S099, S109, S113, S137
M. clathrata	F	“spotted”	S035, S036, S041, S042, S047, S048, S053, S054, S057, S059
M. clathrata	F	“joined”	S033, S034, S037, S038, S039, S040, S051, S055, S056, S058
M. clathrata	M	“spotted”	S003, S006, S021, S022, S023, S024, S025, S026, S027, S031
M. clathrata	M	“joined”	S002, S004, S005, S009, S011, S015, S016, S017, S018, S028

Table S1: The specimen numbers of all wings used in the Mann-Whitney-Wilcoxon Test.

Species	Sex	W	p-value
M. australasiella	F	92.5	0.0015**
M. australasiella	M	99	0.0002***
M. clathrata	F	41.5	0.5437
M. clathrata	M	37	0.3438

Table S2: The relationship between wing pattern and wing length in the two species of *Moerarchis* examined here.
